# Pharmacological Blockade of PPARα Exacerbates Inflammatory Pain-Related Impairment of Spatial Memory in Rats

**DOI:** 10.3390/biomedicines9060610

**Published:** 2021-05-27

**Authors:** Jessica C. Gaspar, Catherine Healy, Mehnaz I. Ferdousi, Michelle Roche, David P. Finn

**Affiliations:** 1 Pharmacology and Therapeutics, National University of Ireland Galway, H91 W5P7 Galway, Ireland; jeccgaspar@gmail.com (J.C.G.); c.healy22@nuigalway.ie (C.H.); m.ferdousi1@nuigalway.ie (M.I.F.); 2 Galway Neuroscience Centre, National University of Ireland Galway, H91 W5P7 Galway, Ireland; michelle.roche@nuigalway.ie; 3 Centre for Pain Research, National University of Ireland Galway, H91 W5P7 Galway, Ireland; 4 Physiology, National University of Ireland Galway, H91 W5P7 Galway, Ireland

**Keywords:** peroxisome-proliferator activated receptor, cognition, anxiety, spatial memory, complete Freund adjuvant, PEA, OEA, dorsal hippocampus

## Abstract

Peroxisome proliferator-activated receptors (PPARs) are ligand-dependent transcription factors that exist in three isoforms: PPARα, PPARβ/δ and PPARγ. Studies suggest that the PPAR signalling system may modulate pain, anxiety and cognition. The aim of the present study was to investigate whether endogenous signalling via PPARs differentially modulates innate anxiety responses and mnemonic function in the presence and absence of inflammatory pain. We examined the effects of intraperitoneal administration of GW6471 (PPARα antagonist), GSK0660 (PPARβ/δ antagonist), GW9662 (PPARγ antagonist), and *N*-palmitoylethanolamide (PEA) on rat behaviour in the elevated plus maze (EPM), open field (OF), light-dark box (LDB), and novel object recognition (NOR) tests in the presence or absence of chronic inflammatory pain. Complete Freund’s Adjuvant (CFA)-injected rats exhibited impaired recognition and spatial mnemonic performance in the NOR test and pharmacological blockade of PPARα further impaired spatial memory in CFA-treated rats. *N*-oleoylethanolamide (OEA) levels were higher in the dorsal hippocampus in CFA-injected animals compared to their counterparts. The results suggest a modulatory effect of CFA-induced chronic inflammatory pain on cognitive processing, but not on innate anxiety-related responses. Increased OEA-PPARα signalling may act as a compensatory mechanism to preserve spatial memory function following CFA injection.

## 1. Introduction

Peroxisome proliferator-activated receptors (PPARs) are ligand-dependent transcription factor members of the nuclear hormone superfamily of receptors. There are three PPAR isoforms: PPARα, PPARβ/δ and PPARγ [1]. Endogenous ligands at PPARs include fatty acids [2] and N-acylethanolamines (NAEs) such as anandamide (AEA) [3,4], *N*-palmitoylethanolamide (PEA) [5], and *N*-oleoylethanolamide (OEA) [6]. PPARs are involved in a diverse array of physiological processes and are drug targets for treating diabetes [7] and dyslipidemia [8]. Moreover, studies suggest that the PPAR signalling system may modulate pain [9], anxiety [10] and cognition [11,12,13,14,15]. PPARs also regulate inflammatory processes and PPAR agonists have anti-inflammatory effects in models of chronic inflammation [16,17].

While there are some important differences between the PPAR subtypes in terms of tissue expression, endogenous and synthetic ligands and physiological roles, all three subtypes of PPARs are expressed in brain regions that are commonly implicated in pain, anxiety and cognition such as the amygdala [18], PFC [18,19,20], hippocampus [10,19] and periaqueductal grey (PAG) [21]. However, few studies have investigated the role of PPARs in anxiety and cognition. Endogenous ligands at PPARs have been shown to be increased in response to stress or anxiety [22,23]. Meanwhile, levels of OEA are significantly lower in patients with post-traumatic stress disorder (PTSD) compared to controls [24]. Additionally, the administration of PEA attenuated aggressiveness in a social isolation model of PTSD in mice [25]. Fernandez et al. (2009) [26] revealed that naringin, a bioflavonoid isolated from citrus fruits and an endogenous ligand of PPARγ, had anxiolytic and antidepressant effects. Another study indicated that seipin knockout (Seipin-KO) male mice displayed anxiety- and depression-like behaviour associated with decreased levels of PPARγ mRNA and protein in the hippocampus and cortex [27], and the administration of the PPARγ agonist rosiglitazone attenuated the anxiety-like behaviour in male Seipin-KO mice. PPARγ genetic deletion had anxiogenic effects in mice [10]. In this same investigation, the authors showed that systemic and intra-amygdala injections of pioglitazone (PPARγ agonist) reduced stress-induced anxiety-like behaviour in rats and that these effects were blocked by the administration of the PPARγ antagonist GW9662. Rosiglitazone elicited antidepressant and anxiolytic-like behavioural effects in mice and pretreatment with the PPARγ selective antagonist GW9662 blocked the effects of rosiglitazone [28]. Recently, administration of pioglitazone was shown to attenuate harmaline-induced anxiety-like behaviours and spatial learning and memory impairments [29], similar to what was observed with rosiglitazone-treated animals. Likewise, Youssef et al. (2019) [30] have shown that the administration of GW9662 blocked the anxiolytic effect of beta-caryophyllene. In other work, repeated stress decreased PPARγ expression in the amygdala, and treatment with anxiolytics recovered PPARγ expression [31]. Gemma et al. (2004) [32] demonstrated that young and aged rats fed with a diet rich in rosiglitazone had increased freezing duration in a context-induced fear protocol. In addition, the levels of PEA were shown to be increased in the basolateral amygdala (BLA) of fear-conditioned (FC) rats [33]. Notably, our group has recently shown that the pharmacological blockade of PPARα and PPARβ/δ, in the presence of formalin-evoked nociceptive tone, impaired short-term fear-extinction in rats, while the blockade of PPARγ potentiated conditioned fear responding, suggesting that endogenous signalling through these three PPAR isoforms may reduce expression of conditioned fear in the presence of nociceptive tone [15].

Administration or manipulation of the levels of endogenous ligands at PPARs, some of which are substrates for fatty acid amide hydrolase (FAAH), have also been shown to enhance cognitive performance [34,35,36,37,38,39,40,41,42]. A few studies indicate a possible modulatory effect of PPARs on memory and learning processes. Mazzola et al. (2009) [12] have shown that the administration of URB597 (a FAAH inhibitor) enhanced the learning of a passive avoidance test, an effect that was attenuated by the administration of a PPARα antagonist MK886. These authors also demonstrated that the administration of a PPARα agonist WY14643 produced similar effects to those observed with URB597, and that these effects were also blocked by MK886. Also, a study from Campolongo et al. (2009) [43] indicated that the administration of OEA improved learning of passive avoidance and spatial memory tasks when given immediately post-training, and that the actions of OEA were mimicked by the PPARα agonist GW7647 and are absent in PPARα null mice. Recently, Ratano et al. (2017) [44] have shown that the cognitive-enhancing effects of URB597 were dependent on PPARα, as well as CB_1_ receptors and TRPV_1_. Together, these studies indicate a modulatory role of PPAR signalling in memory acquisition and consolidation.

PPARs are also known to modulate pain responses [9]. Previous studies in rodents have shown that the selective activation of PPARα [20,45,46,47,48], PPARβ/δ [49,50], and PPARγ [51,52,53,54,55,56] has antinociceptive effects. The administration of PEA, an agonist at PPARs, also has antinociceptive effects in rodents [45,48,57,58,59,60,61,62,63,64,65,66] and in humans [67,68]. Likewise, administration of the endogenous PPAR ligand OEA, and OEA-derived compounds, diminishes nociceptive behaviour [69,70,71]. Pain can impact significantly on both anxiety [72,73,74,75,76,77,78,79] and cognition [80]. Moreover, co-morbidity of chronic pain with anxiety disorders and/or cognitive impairment is highly prevalent [81,82,83]. Recently, our group reported that, in the presence of a nociceptive tone, the systemic administration of PPARα and PPARβ/δ antagonist impaired short-term fear-extinction in rats and the blockade of PPARγ potentiated conditioned fear responding [15].

The aim of the present study was to test the hypothesis that endogenous signalling via PPARs differentially modulates innate anxiety responses and mnemonic function in the presence and absence of inflammatory pain. Selective antagonists were used to block the actions of endogenous ligands at PPARs. Thus, we examined the effects of intraperitoneal administration of GW6471 (PPARα antagonist), GSK0660 (PPARβ/δ antagonist), GW9662 (PPARγ antagonist), and PEA (agonist at the three isoforms), on rat behaviour in the elevated plus maze (EPM), open field (OF), light-dark box (LDB), and novel object recognition (NOR) tests in the presence or absence of chronic inflammatory pain induced by intra-plantar injection of complete Freund’s adjuvant (CFA).

## 2. Materials and Methods

### 2.1. Animals

Experiments were carried out on a total of 80 adult male Sprague-Dawley rats (230–250 g on arrival; Envigo UK, Bicester, UK). The animals were maintained at controlled temperature (22 ± 2 °C) and humidity (45–55%) under standard lighting conditions (12:12 h light-dark cycle, lights on from 07.00 h). All experiments were carried out during the light phase. Food and water were available ad libitum. The experimental procedures were approved by the Animal Care and Research Ethics Committee, National University of Ireland, Galway (Project ID: 15-Feb-01, date of approval: 6 May 2015). The work was carried out under license from the Health Products Regulatory Authority in the Republic of Ireland (Project Authorisation: AE19125_P028; Date of Approval: 7 August 2015) and in accordance with EU Directive 2010/63.

The animal studies are reported in compliance with the ARRIVE guidelines for preclinical research described by Percie Du Sertie et al. (2020) [84].

### 2.2. Drugs

GW6471, GSK0660, GW9662 and PEA (all obtained from Tocris Bioscience, Bristol, UK) were dissolved in a 1:1:8 (ethanol:cremophor:saline) vehicle solution. The doses of GW6471 (2 mg/kg), GSK0660 (1 mg/kg) and GW9662 (2 mg/kg) were chosen based on studies in the literature demonstrating the efficacy of these three PPAR antagonists in reversing the antinociceptive and neuroprotective effects of PEA [66,85] or pioglitazone [52,56,86], as well as the results published previously by our group [15]. Immunogenic complete Freund’s adjuvant emulsifier (CFA, desiccated Mycobacterium tuberculosis in an 85% mineral oil, 15% mannide monooleate suspension, Sigma-Aldrich, Dublin, Ireland) was used to induce a chronic inflammatory pain state [87]. Rats received a single 100 µL intraplantar injection of CFA (1 mg/mL) into the right hind paw, under brief isoflurane anaesthesia (3% in 0.8 L/min O_2_). Control rats underwent intraplantar needle insertion to the right hind paw, also under isoflurane anaesthesia.

### 2.3. Experimental Design

**Figure 1:**
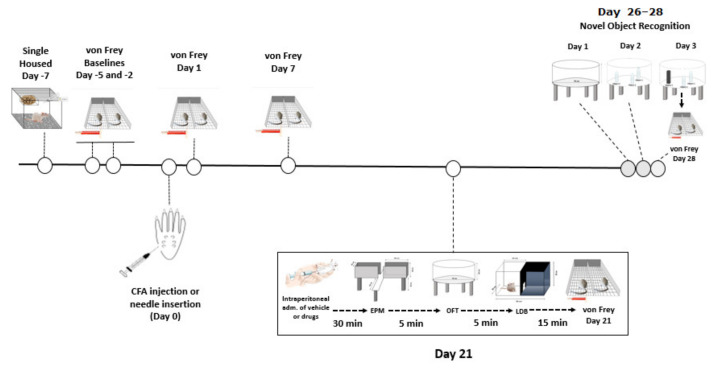
Graphical representation of the experimental design.

The animals were housed initially in groups of three and allowed 4 days of habituation upon arrival. Five days after arrival, seven days before Complete Freud Adjuvant (CFA) injections, the rats were singly housed (the experimental design is summarized in Figure 1). The experimental unit was an individual rat (for behavioural tests) or brain tissue from each individual rat (LC-MS/MS). On day 7 and 10 after arrival (5 and 2 days before CFA injection), the baseline paw withdrawal thresholds for mechanical sensitivity were determined using the von Frey test. The rats were placed in one of the six chambers of the von Frey apparatus where they were allowed to habituate for 15 min. Then, the rats received 9 stimulations in each paw using the von Frey filaments according to the up-and-down method described by Dixon [88]. All responses were recorded and analysed afterwards. On day 12 after arrival, day 7 after single housing, the rats were pseudo-randomly divided into two groups such that the average baseline paw withdrawal thresholds did not differ between the two groups: the rats allocated to the CFA-treated group received a 100 μL intra-plantar injection of CFA into the right hind paw under isoflurane (2–3% in O_2_, 0.8 L/min) anaesthesia, while animals in the No-CFA group underwent needle insertion alone into the right hind paw under isoflurane anaesthesia. After injections, the rats were immediately returned to their home cages. On the following day, the first post-CFA paw withdrawal threshold data collection took place, and another set of data was collected on day 7 post-CFA injection.

On day 21 post-CFA, the rats were tested for anxiety-related behaviour. The animals received an intraperitoneal injection of GW6471 (2 mg/kg), GSK0660 (1 mg/kg), GW9662 (2 mg/kg), PEA (2 mg/kg) or vehicle in an injection volume of 3 mL/kg. Thus, the experimental groups were: Vehicle-No CFA, GW6471-No CFA, GSK0660-No CFA, GW9662-No CFA, PEA-No CFA, Vehicle-CFA, GW6471-CFA, GSK0660-CFA, GW9662-CFA, PEA-CFA. Rats were randomly assigned to treatment groups and the sequence of testing was randomised to avoid confounding effects of test group order. Thirty minutes after injections, the rats underwent a series of anxiety tests: they were initially placed in the elevated plus maze (EPM) arena for 5 min, followed by the open field (OF) test for 5 min, and then the light-dark box (LDB) test also for 5 min. After all the anxiety tests were completed, the rats were again placed in the von Frey apparatus for a 15 min habituation followed by an assessment of paw withdrawal thresholds. The rats were returned to their home cages after von Frey testing. The time of testing post-injection was chosen based on a previous work published by our research group showing that these antagonists crossed the blood-brain barrier to reach brain tissue and exert pharmacological effects 30 min following intraperitoneal injection [15].

On day 26, post-CFA injection, we initiated the novel object recognition test (NOR) protocol. Briefly, on the first day of the protocol (day 26 post-CFA), rats were allowed to explore the NOR arena, which at this point had no objects, in a habituation trial for 10 min. On the next day (day 27 post-CFA), the rats were exposed to the familiarization phase, in which they were allowed to explore freely for 5 min in the arena where three plastic bottles filled with water were placed. This protocol was repeated 3 times, with 5-min breaks between exposures. After the third exposure, the rat was returned to its home cage. On the test day (day 28 post-CFA), the animals received an intraperitoneal injection of GW6471 (2 mg/kg), GSK0660 (1 mg/kg), GW9662 (2 mg/kg), PEA (2 mg/kg) or vehicle in an injection volume of 3 mL/kg. Rats were pseudo-randomly re-assigned to drug treatment groups relative to the treatments they received prior to anxiety testing on day 21 post-CFA using the Latin Square Randomisation method. Thirty minutes after administration of drugs, the rats were placed in the NOR arena for 5 min, with one of the plastic bottles replaced by a squared plastic structure (novel object). The time spent exploring the familiar water-filled bottles and the novel object was recorded and later analysed. Again, when the NOR test was finished, the rats were placed in the von Frey apparatus for a 15 min habituation followed by the sixth and final paw withdrawal threshold test. After the von Frey data collection, rats were euthanized by live decapitation and the brains were harvested, snap-frozen on dry ice, and stored at −80 °C. Sample sizes were based on previous studies with our laboratory and others that followed a similar design and had similar outcome measures. Final sample sizes were *n* = 8 per group for the EPM and OF, and *n* = 7–8 per group for LDB, von Frey test, NOR and LC-MS/MS. In the LDB, 3 animals failed to move from the initial chamber (light chamber) to the dark chamber (one in each of the following groups: No CFA-Vehicle, No CFA-PEA, CFA-GW6471). In the von Frey test, one animal (in the No-CFA GW6471 group) did not respond to the filaments at baseline and Day 1. In the NOR, 2 rats were deemed outliers for discrimination index and spatial discrimination index (1 in No CFA-Vehicle, and 1 in CFA-GW6471), and 6 rats for preference index (1 in each of No CFA-Vehicle, CFA-Vehicle, CFA-GW6471, No CFA-GSK0660, No CFA-PEA, CFA-PEA), based on being >2 × standard deviations from their group means. For LC-MS/MS, *n* = 7 per group for all groups and all analytes except for PEA in the No CFA-Vehicle group where *n* = 8.

### 2.4. Behavioural Tests

#### 2.4.1. Von Frey Test for Mechanical Hypersensitivity

The von Frey test apparatus comprised a six-chambered arena made of clear Perspex front and back walls and white chipboard lateral walls. The dimensions of the chambers were such that rats could move freely (14 cm × 20 cm × 25 cm). A Perspex lid with air-holes was placed on top of the arena during the habituation and testing periods. In all experiments, the arena was placed on a raised wire-mesh flooring so that the experimenter (who was blind to treatment) could access the hind paws of the rats from below. Six rats were tested per session, and the arena was thoroughly cleaned between each session using 70% ethanol. Rats received an initial habituation period of 15 min during which they were placed in individual chambers of the arena. The baseline withdrawal thresholds were acquired twice, on days −5 and −2, before CFA intraplantar injection on day 0. For the statistical analysis, the average of both baselines was used. We applied the up-and-down method described by Dixon [88]. In this method, the rats receive a maximum of 9 nylon von Frey filament stimulations (Touch Test Sensory Evaluator #58011, Stoelting, IL, USA), starting with the 2 g filament. Each filament was applied only once, perpendicular to the plantar surface of the hind paw, targeting the area at the base of the third and fourth digits (from medial to lateral) according to the previous protocol used by the group, with sufficient force to cause slight buckling of the filament, for approximately 6 s or until a positive response was observed. A positive response was recorded if flinching, licking or withdrawal of the paw occurred on application of the filament or immediately after removal of the filament. Filaments were applied to both the left and right hind paws (alternating between paws). First, the thresholds for the contralateral paws of all six rats were collected, followed by the thresholds for the ipsilateral paws. If a positive response was observed using the 2 g filament, filaments of lower weights (down) were applied in descending order until no positive responses were observed. If there was no response using the 2 g filament, filaments of higher weights (up) were applied in ascending order until a positive response was observed. In addition, the following approach was followed: positive responses lead to stimulation with the next lowest filament, and negative responses lead to stimulation with the next highest filament, until a total of 9 stimuli were applied. These nine digits generate a code that is associated to a constant (*κ*) detailed by Dixon [88]. The final value for the paw withdrawal threshold is calculated using the formula: 10^[(log (last hair)+*κ*)^^∗0.3].^

The withdrawal thresholds of CFA-treated animals were compared to the no-CFA control group and the effect of the treatments on CFA or no-CFA animals compared to the vehicle-treated counterparts (control) was also analysed.

#### 2.4.2. Elevated Plus Maze

The EPM arena consisted of a white wooden plus-shaped maze elevated 50 cm from the room floor with two arms enclosed by walls (30 cm) and two open arms; the floor was covered in a black rubber material. Each arm was 50 cm in length and 10 cm in width and the arms were interconnected by a central platform. A video camera was positioned over the maze and the light levels were fixed at 60 lux in the open arms and 25 lux in the closed arms, according to the protocol previously used by our group. The rat behaviour was recorded and analysed using a computerized video tracking system (EthoVision^®^ XT11.5, Noldus, The Netherlands) for a 5 min period. The EPM was cleaned between animals with 70% Ethanol. Reduced time spent in the open arm(s) was used as an experimental index of anxiety. Entries in arms were defined as entry of the rat’s centre of gravity into the arms (centre point on the body). The time spent in either the open or closed arms by CFA-treated animals was compared to the no-CFA control group and the effect of the treatments on CFA or no-CFA animals compared to the vehicle-treated group (control) was also analysed.

#### 2.4.3. Open Field Test

Behaviour in the open field was assessed once according to the experimental design described above in Figure 1. The rats were placed into a brightly lit (200 lux) open field environment (diameter 75 cm and 40 cm high walls, of reflective aluminium walls and floor). A camera positioned 35 cm above the floor of the arena allowed for behaviour to be captured, recorded and assessed using a computerized video tracking system (EthoVision^®^ XT11.5, Noldus, The Netherlands) for a 5 min period. The open field was cleaned between animals with 70% ethanol. The behavioural assessment included locomotor activity (total distance moved) and time spent (seconds) in the centre zone (45 cm diameter). Reduced time spent in the centre zone is interpreted as anxiety-related behaviour. The time spent in either the centre or periphery of the arena and the total distance moved by CFA-treated animals were compared to the no-CFA control group and the effect of the treatments on CFA or no-CFA animals compared to the vehicle-treated animals (control) was also analysed.

#### 2.4.4. Light-Dark Box

Behaviour in the light-dark box was assessed once according to the experimental design described above in Figure 1. The rats were placed into a 30 cm × 30 cm × 30 cm Perspex chamber divided into two compartments that were connected by an entrance. One of the compartments is defined as light-chamber and was brightly illuminated (150 lux) while the other was called dark-chamber (0 lux at the corners and 5 lux next to the passage door). A camera was positioned below the arena and the behaviours were recorded and later assessed using a computerized video tracking system (EthoVision^®^ XT11.5, Noldus, The Netherlands) for a 5 min period. The light-dark box arena was cleaned between animals with 70% ethanol. The behavioural assessment included locomotor activity (total distance moved), time spent (seconds) in each of the chambers and the number of entrances in the dark chamber. Reduced time spent in the light compartment is interpreted as anxiety-related behaviour. Therefore, the time spent in either the light or dark chamber of the arena, the latency to move from the light to the dark chamber, and the total distance moved by CFA-treated animals were compared to the no-CFA control group, and the effect of the treatments on CFA or no-CFA animals compared to the vehicle-treated animals (control) was also analysed.

#### 2.4.5. Novel Object Recognition

**Figure 2:**
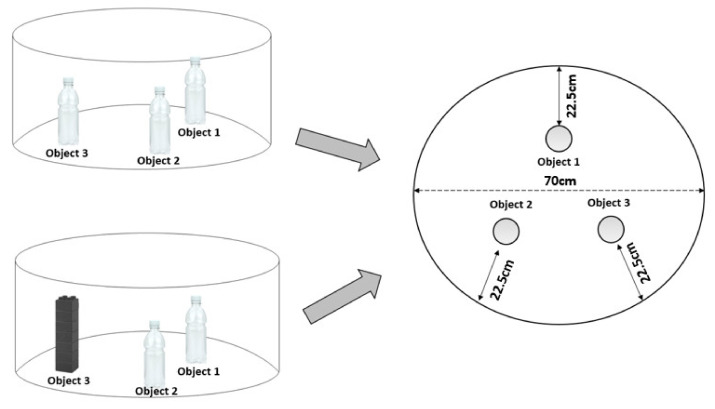
Graphical representation of the NOR arena. The left-top image represents the organisation of the object on the sample (familiarisation) day. The left-bottom image represents the organisation of the objects on the test day (novel object in the position of object 3). The right image shows the equal distances of the object to the wall of the circular arena.

Testing was carried out in the same circular arena used for the open field test. In all experiments, the arena was illuminated by constant light intensity of 100 ± 10 lux at floor level of the arena. A camera positioned above recorded the whole test for subsequent analysis. The objects used included 500 mL unlabelled transparent thin plastic polyethylene terephthalate Coca-Cola^®^ bottles filled with water, and an abstract plastic structure with a base 5 cm × 5 cm and height 16 cm constructed from a mixture of green, white and blue toy blocks (Playskool Clipo^®^ blocks; Pawtucket, RI, USA). In all cases, the objects had no apparent natural significance to the rats and were secured to the base of the arena with white tack such that they were difficult to displace. Animals were habituated to the arena in the absence of objects for 10 min on day 1 (see Figure 2). On the second day (familiarisation), three identical objects (Coca-Cola^®^ bottles; Atlanta, GA, USA) were placed in the arena 16 cm from points on the perimeter of the circular arena. The rat was allowed to freely explore the arena and objects three times for 5 min, with 5 min intervals between exposures. After this exposure, the animal was removed from the arena and returned to its home cage. On the following day (test), one of the objects was replaced with a novel object (abstract plastic structure constructed with a mixture of green, white and blue toy blocks). The animal was allowed to freely explore the arena and objects for a period of 5 min and then returned to its home cage. The arena was cleaned with 70% ethanol and faecal pellets were removed between each exposure to remove odours and olfactory cues. Exploration of an object was defined as sniffing the object, rearing against the object or having the head directed towards the object within 2 cm of the object. In all cases, the experimenter rating the behaviour was blind to the experimental treatment of the rat (CFA or drug). Ethovision^®^ XT11.5; Wageningen, the Netherlands) as also used to track the distance (in cm) moved by the animal during testing. The position of the novel object was alternated between rats in order to minimise potential confounding effects related to orientation biases. Three indices were calculated in order to assess NOR results: (1) the preference index defined as the time spent preferentially with the novel object in relation to the time spent with the familiar object in the same position; (2) the discrimination index defined as the time spent with the novel object in relation to the time spent with the familiar objects in the test day; (3) the spatial discrimination index, defined as the time spent in the location of the new object in relation to the time spent in the same location in the familiarisation phase (the equations used in each of these rations can be seen in Table 1). The time spent exploring the objects and the arena where the objects were located by CFA-treated animals were compared to the no-CFA control group, and the effect of the treatments on CFA or no-CFA animals compared to the vehicle-treated animals (control) was also analysed.

### 2.5. Liquid Chromatography—Tandem Mass Spectrometry (LC-MS/MS)

Tissue extraction was carried out using the following method: the previously dissected brain region samples (dorsal hippocampus and entorhinal cortex) were homogenised for 4–6 s in a mixture containing 200 μL of deuterated internal standards for endocannabinoids (0.48 nmol/50 ng of 2-AG-d8 and 0.014 nmol/2.5 ng of AEA-d8) and NAEs (0.015 nmol/2.5 ng of OEA-d4 and 0.016 nmol/2.5 ng of PEA-d4). The final volume was made up to 275 μL by adding 100% acetonitrile using an ultrasonic homogeniser/sonicator (Mason, Dublin, Ireland). Deuterated and non-deuterated endocannabinoids were purchased from Cayman Chemicals (Cambridge, Biosciences, UK).

Samples were kept on ice during the procedure. The homogenates were centrifuged at 14,000 RPM for 15 min at 4 °C (Hettich centrifuge Mikro 22R, Tuttlingen, Germany). Immediately after, the supernatant was collected and 40 μL was transferred to a HPLC vial. The standard curve was constructed using serial 1/4 dilution by adding 25 µL of a mixture of non-deuterated endocannabinoids (12.5 ng for PEA, OEA and AEA + 125 ng for 2-AG) to tube #10, which contained 75 µL of acetonitrile, vortex-mixing, then collecting 25 µL and transferring to the next tube (#9) containing 75 µL acetonitrile. The process was repeated until tube #1, when 25 µL of the final volume was discarded, in order to keep the volumes between tubes consistent. Thus, all 10 tubes had 75 µL of a mixture of endocannabinoids. All standard curve tubes were spiked with 200 µL of deuterated endocannabinoid mixture (2.5 ng deuterated PEA, OEA and AEA and 50 ng deuterated 2-AG as internal standards) to give a final volume of 275 µL per tube. 40 μL of each standard was transferred to a corresponding HPLC vial. A double blank (100% acetonitrile) was also included in between each standard point during the run to minimise the risk of analyte carryover from standard to standard at the upper range of the curve and five double blanks were included after the highest concentration point on the curve to avoid carryover onto the samples. A quality control (QC) sample was prepared from the whole rat brain homogenate was included with each run to allow for monitoring of inter-runs variability. The QC was added after all the samples, at the end of the run.

The mobile phase consisted of (A) high-pressure liquid chromatography grade water and (B) acetonitrile, both containing 0.1% *v/v* formic acid. At time 0, solvent ratios were (55:45) (*v/v*) respectively, remaining as such for 1 min and then ramped up linearly to 100% solvent B by minute 5. The volume of solvent B (100%) remained as such until minute 12, whereby the sample run was completed. At 12.1 min, a five-minute equilibration phase occurred between each run to facilitate the return of the gradient to initial conditions specified at time 0.

Quantitation of each analyte was performed by determining the peak area response of each target analyte against its corresponding deuterated internal standard. This ratiometric analysis was calculated using Masshunter Quantitative Analysis Software B.07.00 (Agilent Technologies Ltd., Cork, Ireland). The amount of analyte in unknown samples was calculated from the analyte/internal standard peak area response ratio using a 10-point calibration curve constructed from a range of concentrations of the non-deuterated form of each analyte and a fixed amount of deuterated internal standard. The values obtained from the Masshunter Quantitative Analysis Software are initially expressed in ng per mg of tissue by dividing the weight of the punched tissue. To express values as nmol or pmols per mg the corresponding values are then divided by the molar mass of each analyte expressed as ng/nmole or pg/pmole.

### 2.6. Statistical Analysis

The SPSS 24.0 statistical package was used to analyse data. Normality was assessed using Shapiro-Wilk test and homogeneity of variance was checked using Levene’s test. Behavioural data were analysed using a two-factor analysis of variance (Two-way ANOVA), with factors being CFA injection and treatment, or repeated measures ANOVA when appropriate (e.g., when the data were analysed and presented in time bins) and the effect size of ANOVA was evaluated by η^2^. Post hoc pairwise comparisons were made with Student-Newman-Keuls test when appropriate and effect size was evaluated by Cohen’s d coefficient (d). If data were found to be non-parametric, three transformations were applied, in this order: square root of the data, log of the data, and ranking of the data. In cases where the data, after the transformations, still failed to pass the normality and/or the homogeneity of variance tests, a non-parametric analysis was applied. Non-parametric data were analysed using Kruskal Wallis analysis of variance and post hoc analysis performed using Dunn’s test when appropriate. When repeated measures were non-parametric, data were analysed using Friedman’s and Kruskal Wallis tests followed by Dunn’s post hoc or with Mann Whitney adjusted with Bonferroni-Holm corrections tests if applicable. Data were considered significant when *p* < 0.05. Data are expressed as group means ± standard error of the mean (SEM) when parametric and as median with interquartile range and min/max values when non-parametric is unless otherwise stated.

## 3. Results

### 3.1. CFA Induced Mechanical Hypersensitivity Measured by von Frey Testing

**Figure 3:**
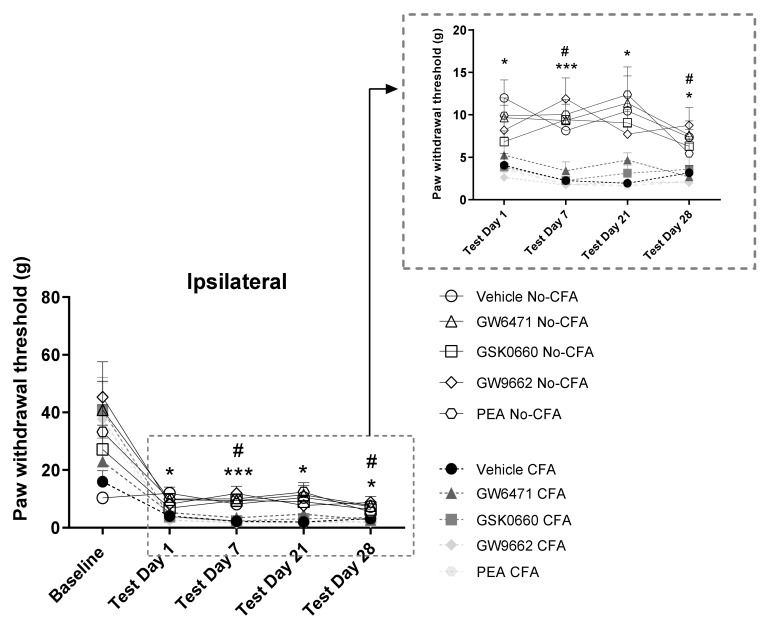
Effects of systemic administration of vehicle, selective PPARα (GW6471), PPARβ/δ (GSK0660) and PPARγ (GW9662) antagonists, and PEA on mechanical hypersensitivity in CFA-injected (CFA) and control (No-CFA) rats. Post hoc testing indicated significantly lower paw withdrawal thresholds in CFA vehicle-treated animals on days 1, 7, 21, and 28 (* *p* < 0.05 and *** *p* < 0.001, vs. No-CFA Vehicle) in the ipsilateral paw (B). The test also indicated lower paw withdrawal thresholds in CFA GW9662 and PEA-treated animals compared to their No-CFA counterparts (# *p* < 0.05 vs. No- CFA GW9662, days 7 and 28; # *p* < 0.05 vs. No-CFA PEA, days 7 and 21). The von Frey data, which were non-parametric, are presented in timeline graphs as means ± S.E.M. for presentation/readability purposes (*n* = 7–8 rats per group).

Intra-plantar injection of CFA into the right hind paw produced robust nociceptive behaviour as evidenced by the lower ipsilateral hind paw withdrawal thresholds measured in the von Frey testing (baseline [χ^2^ (9) = 8.236, *p* > 0.05] η^2^ = 0.015; day 1 [χ^2^ (9) = 35.069, *p* < 0.001] η^2^ = 0.001; day 7 [χ^2^ (9) = 48.980, *p* < 0.001] η^2^ = 0.008; day 21 [χ^2^ (9) = 51.601, *p* < 0.001] η^2^ = 0.001; day 28 [χ^2^ (9) = 39.580, *p* < 0.001] η^2^ = 0.024). Post hoc Dunn’s test indicated significantly lower paw withdrawal thresholds in CFA vehicle-treated animals compared to their No-CFA counterparts in days 1, 7, 21, and 28 (CFA Vehicle vs. No-CFA Vehicle, * *p* < 0.05 and ** *p* < 0.01; d = 0.105, 0.685, 0.813, 0.438; Figure 3). The test also indicated lower paw withdrawal thresholds in CFA GW9662 and PEA-treated animals compared to their No-CFA counterparts (CFA GW9662 vs. No-CFA GW9662, days 7 and 28, ^#^ *p* < 0.05, d = −0.285, −0.271; CFA PEA vs. No-CFA PEA, days 7 and 21, ^#^ *p* < 0.05, d = −0.138, 0.068).

### 3.2. No Effect of PPAR Antagonists or PEA on Anxiety-Related Behaviour in the Elevated Plus Maze (EPM) in CFA or No-CFA Treated Rats

**Figure 4:**
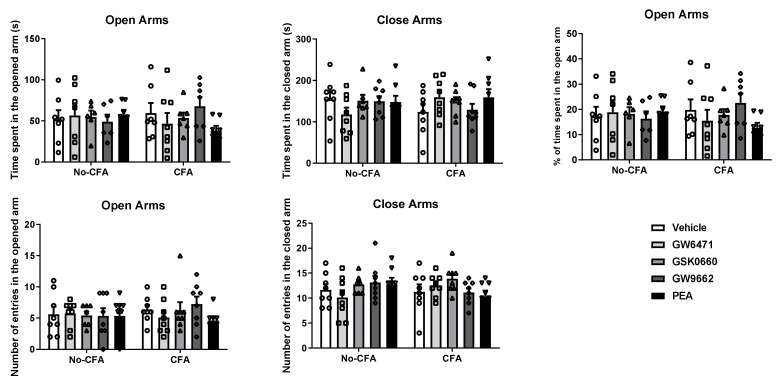
Effects of systemic administration of vehicle, selective PPARα (GW6471), PPARβ/δ (GSK0660) and PPARγ (GW9662) antagonists, and PEA on anxiety-related behaviours in the EPM test in CFA-injected (CFA) and control (No-CFA) rats. Data are expressed as means ± SEM (*n* = 8 rats per group). The symbols represent each individual data point.

The systemic administration of PPAR antagonists or PEA did not have any effect on the behaviour of CFA or No-CFA treated animals in the EPM test (Figure 4). Intra-plantar injection of CFA did not affect the behaviour of the animals in the test.

### 3.3. No Effect of PPAR Antagonists or PEA on Anxiety-Related Behaviour in the Open Field in CFA or No-CFA Treated Rats

**Figure 5:**
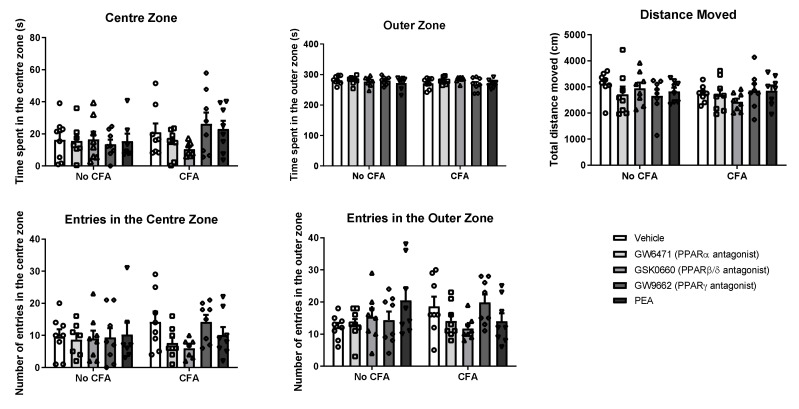
Effects of systemic administration of vehicle, selective PPARα (GW6471), PPARβ/δ (GSK0660) and PPARγ (GW9662) antagonists, and PEA on exploratory and anxiety-related behaviours in the OF test in CFA-injected (CFA) and control (No-CFA) rats. Data are expressed as means ± SEM (*n* = 8 rats per group). The symbols represent each individual data point.

The systemic administration of PPAR antagonists or PEA did not have any effect on the behaviour of CFA or No-CFA treated animals in the open field test (Figure 5). Intra-plantar injection of CFA did not affect the behaviour of the animals in the test.

### 3.4. Effects of PPAR Antagonists on Anxiety-Related Behaviour in the Light-Dark Box (LDB) in CFA-Treated Rats

**Figure 6:**
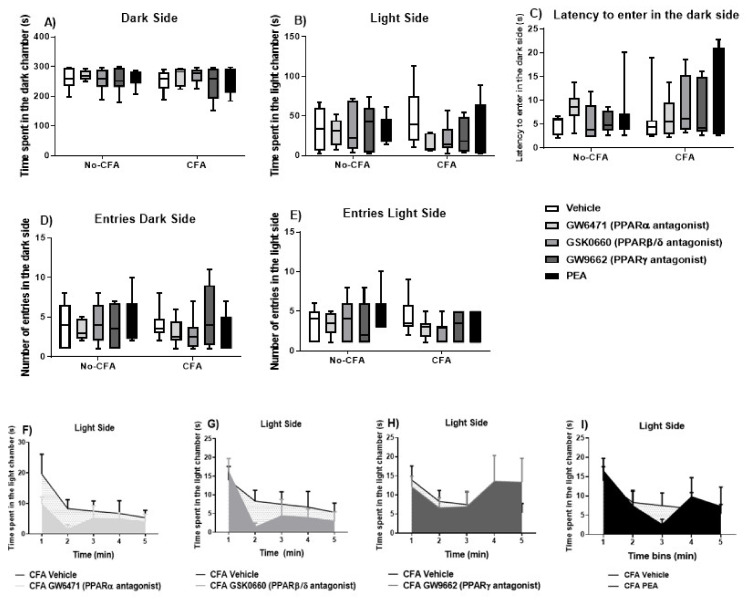
Effects of systemic administration of vehicle, selective PPARα (GW6471), PPARβ/δ (GSK0660) and PPARγ (GW9662) antagonists, and PEA on anxiety-related behaviours in the LDB test in CFA-injected (CFA) and control (No-CFA) rats. Data are expressed as median with interquartile range and min/max (**A**–**E**) or means ± SEM (**F**–**I**) (*n* = 7–8 rats per group). In the CFA-injected groups, the area under the curve (AUC) analysis indicated a trend for a decrease in time spent in the light side in GW6471-treated (*p* = 0.075; F) and GSK0660-treated (*p* = 0.07; G) rats compared to vehicle counterparts. The AUC analysis was done on all groups together but are presented separately for presentation/readability purposes. Data are presented as means ± S.E.M. (*n* = 7–8 rats per group).

The systemic administration of PPAR antagonists or PEA did not have any significant effect on the behaviour of the animals in the time spent in the dark side (χ^2^ (9) = 5.060, *p* > 0.05; Figure 6A), latency to enter the dark side (χ^2^ (9) = 10.382, *p* > 0.05; Figure 6C), number of entries into the light side (χ^2^ (9) = 11.067, *p* > 0.05; Figure 6D), or number of entries into the dark side (χ^2^ (9) = 12.610, *p* > 0.05; Figure 6E) in the light-dark box test. Repeated measures ANOVA revealed a significant effect of time on the time spent in the light side (F (4, 349) = 11.51, *p* < 0.001; Figure 6B). An analysis of the area under the curve indicated a trend for a decrease in time spent in the light side in CFA GW6471-treated (*p* = 0.075; Figure 6F) and in CFA GSK0660-treated (*p* = 0.07; Figure 6G) rats compared to CFA vehicle-treated rats.

### 3.5. PPARα Antagonist Impairs Spatial Memory Rats in the NOR Test in CFA-Treated Rats

**Figure 7:**
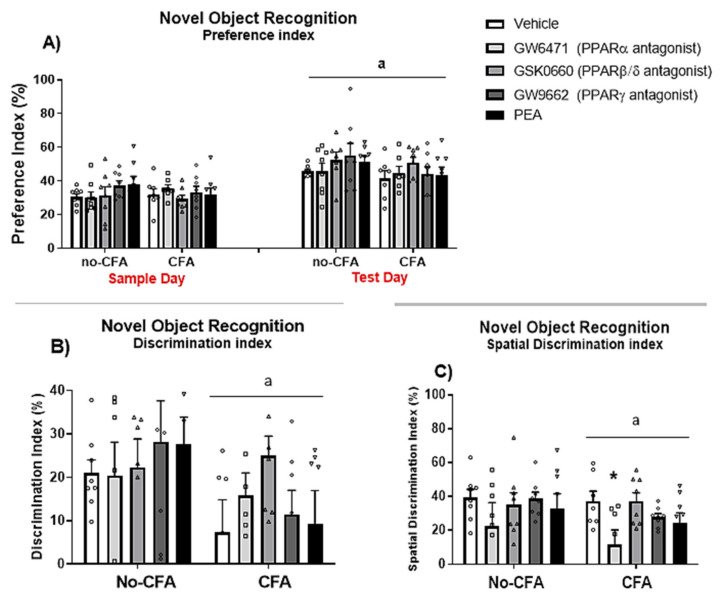
Effects of systemic administration of vehicle, selective PPARα (GW6471), PPARβ/δ (GSK0660) and PPARγ (GW9662) antagonists, and PEA on behaviour in the NOR test in CFA-injected (CFA) and control (No-CFA) rats. Two-way ANOVA revealed an effect of day (^a^ *p* < 0.001) on the percentage of time spent exploring the novel object compared to object 3 (i.e., preference index = TObject3 or Novel Object/(TObject 1 + TObject 2/2) + TObject3 or Novel Object multiplied by 100; (**A**). CFA injection (^a^ *p* < 0.05; (**B**,**C**)) was also shown to have an effect on the discrimination index (Discrimination index = TNovel object/(TObject1 + TObject2/2) + TN multiplied by 100) and on the spatial discrimination index (Spatial discrimination index = TNovel object-TObject3/TNovel object + TObject3 multiplied by 100). GW6471 significantly reduced the spatial discrimination index compared to vehicle-treated CFA rats (* *p* < 0.05, vs. CFA-Vehicle; (**C**)). Data are expressed as means ± SEM (*n* = 7–8 rats per group). The symbols represent each individual data point.

The results show a significant effect of day [F (1, 140) = 50.469, ^a^ *p* < 0.001; η^2^ = 0.248] on the novel object preference index (percentage of time spent exploring the location of the novel object/object 3; (Figure 7A). However, there were no significant effects of treatment [F (4, 140) = 0.772, *p* > 0.05; η^2^ = 0.015], CFA [F (1, 140) = 2.237, *p* > 0.05; η^2^ = 0.011], or interactions of treatment x CFA [F (4, 140) = 0.820, *p* > 0.05; η^2^ = 0.016], treatment x day [F (4, 140) = 0.475, *p* > 0.05; η^2^ = 0.009], CFA × day [F (1, 140) = 0.553, *p* > 0.05; η^2^ = 0.003], and treatment × CFA × day [F (4, 140) = 0.414, *p* > 0.05; η^2^ = 0.008] on the preference index. Importantly, the analysis also revealed a significant effect of CFA [F (1, 62) = 6.006, ^a^ *p* < 0.05; η^2^ = 0.085] on the discrimination index (the percentage of time spent exploring the novel object in relation to the familiar objects in the arena on the test day; Figure 7B). Likewise, there was a significant effect of CFA [F (1, 66) = 5.105, ^a^ *p* < 0.05; η^2^ = 0.048] on the discrimination index for spatial memory which compares the percentage difference in time exploring the novel object on test day in relation to object 3 placed in the same location on sample day (Figure 7C). When the analysis was carried out in CFA vs. non-CFA animals, separately, there were no significant treatment effects on the discrimination index (despite a trend for the PPARβ/δ antagonist GSK0660 to attenuate the CFA-induced reduction in discrimination index), however a significant treatment effect was revealed in the CFA-injected group for the spatial discrimination index [F (4, 33) = 3.239, *p*<0.05; η^2^ = 0.039]. *Post hoc* analysis with Student Newman-Keuls test indicated that the PPARα antagonist GW6471 significantly reduced the discrimination index for spatial memory compared to vehicle-treated CFA rats (* *p* < 0.05, vs. CFA-Vehicle).

### 3.6. Increased OEA Levels in the Ipsilateral Dorsal Hippocampus of CFA-Injected Rats

**Figure 8:**
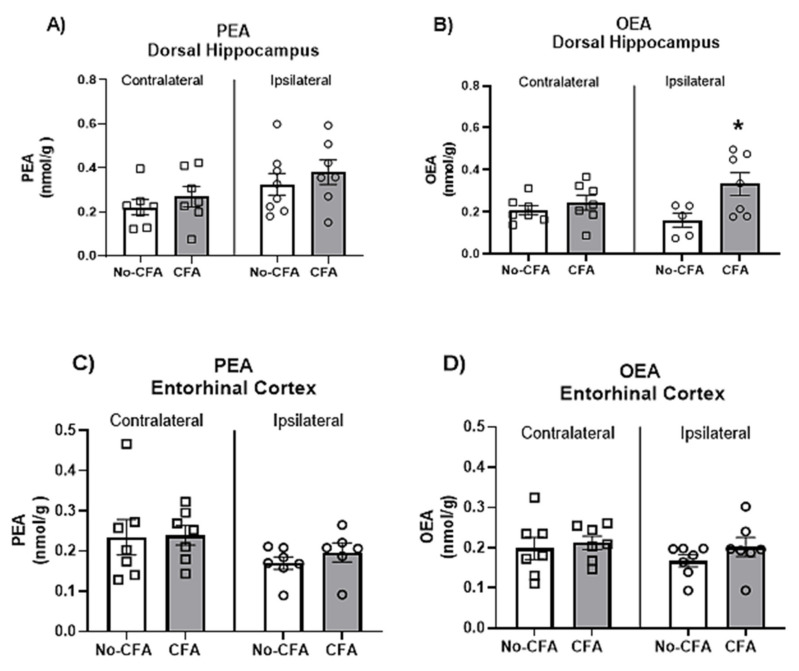
Effects of intraplantar injections of CFA in vehicle-treated animals on the levels of OEA and PEA in the dorsal hippocampus (**A**,**B**) and entorhinal cortex (**C**,**D**). Two-way ANOVA has shown a significant overall effect of CFA on the levels of OEA in the dorsal hippocampus (**B**). Post hoc analysis with Student Newman-Keuls test indicated that OEA levels were significantly higher in the ipsilateral dorsal hippocampus of the CFA-vehicle group compared to No-CFA counterparts (**B**; * *p* < 0.05, vs. no-CFA; d = −0.390). Data are expressed as means ± SEM (*n* = 7–8 rats per group).

In order to further explore the reasons underlying the differential effect of PPARα antagonism on spatial memory depending on the nociceptive tone, the levels of endogenous PPAR ligands (PEA and OEA) in the ipsilateral and contralateral (in relation to the CFA injection which was into the right hind paw) dorsal hippocampus (Figure 8A,B) and entorhinal cortex (Figure 8C,D), two key regions implicated in spatial memory acquisition, were analysed in CFA-vehicle and No-CFA vehicle groups. The results show a significant overall effect of CFA [F (1, 7) = 8.987; η^2^ = 0.022] on the levels of OEA in the dorsal hippocampus (Figure 8B). Post hoc analysis with Student Newman-Keuls test indicated that OEA levels were significantly higher in the ipsilateral dorsal hippocampus of the CFA-vehicle group compared to No-CFA counterparts (* *p* < 0.05, vs. no-CFA; d = −0.390). Two-way ANOVA pointed to an overall side difference (relative to the CFA injection) on the levels of PEA [F (1, 25) = 5.109, *p* = 0.0328; η^2^ = 0.022] in the dorsal hippocampus, but the post hoc analysis with Student Newman-Keuls test did not reveal any further significant differences between relevant groups. CFA injection did result in changes in the levels of OEA or PEA in the entorhinal cortex.

## 4. Discussion

The study described herein investigated the role of PPARs in innate anxiety responses and mnemonic function in the presence and absence of a nociceptive stimulus. A key finding of the work was that CFA-injected rats exhibited impaired recognition and spatial mnemonic performance in the NOR test and that pharmacological blockade of PPARα further impaired spatial memory in CFA-treated rats, but had no effect in No-CFA injected controls. The intraplantar administration of CFA resulted in robust mechanical hypersensitivity in the injected paw on days 1, 7, 21 and 28 post-injection. Injection of GW6471, GSK0660, GW9662 or PEA did not alter pain responses at any of these time points. Several studies have demonstrated the involvement of PPAR signalling in pain responses in acute and chronic models of inflammatory pain [9]. Our results do not show any alteration in CFA-induced pain-related behaviour following systemic administration of PPAR antagonists. These findings are in accordance with Donvito et al. (2017) [89], Mansouri et al. (2015) and our previous results [15], demonstrating that intraperitoneal administration of GW6471 and GW9662 did not affect nociceptive behaviour in the formalin test of tonic, persistent inflammatory pain. Other studies have shown antinociceptive effects of PEA-induced PPAR activation in rodent models of inflammatory [48,59,90] and neuropathic [48,57,63,64,66,71,91] pain, including a study using CFA injections in the temporomandibular nerve [92]. Therefore, our results for PEA diverge to some extent from those reported in the literature, which may relate to differences in our experimental design and methodology compared to other studies.

In the current study, CFA injections did not affect anxiety-related behaviour in the EPM, OF or LDB tests. This result is at odds with other studies showing CFA-induced anxiety. For instance, Parent et al. (2012) [92] have shown that intraplantar injection of CFA induced anxiogenic behaviours in the EPM and OF, but not LDB, tests in rats. Although the authors used the same tests and the same rat strain used herein, they chose different time points post-CFA to run their tests (i.e., day 28–30 vs. day 21 in the present study). Hofmann et al. (2017) [93] have seen CFA-induced anxiety-like behaviour in the LDB and EPM tests with mice 48 h after CFA injection. Other studies have also shown increased anxiety-like behaviours in mice in EPM and OF tests 7 days [94,95], 21 days [96,97], and 6 weeks [98] post-CFA injection. Similarly, intraplantar injections of CFA also resulted in increased anxiety-related behaviour in mice in the place escape/avoidance paradigm (PEAP) and elevated zero maze tests [99]. The injection of CFA into the temporomandibular joint also produced anxiety-like behaviours in the EPM and LDB tests in Wistar rats [100]. In summary, these studies indicate an anxiogenic effect induced by CFA injections. However, differences in the animal model (i.e., rats vs. mice, Sprague-Dawley vs. Wistar) and the time points at which the tests were performed could explain the discrepant results seen in the present study. Several studies have indicated that PPAR signalling is involved in the regulation of anxiety responses. However, in the present study, the systemic administration of PPAR antagonists or PEA did not markedly affect the behaviour of rats in the EPM, OF and LDB tests, although a trend for an anxiogenic effect of the blockade of PPARα and PPARβ/δ in the CFA-injected animals was observed in the LDB test. The findings described here are in accordance with Panlilio et al. (2009) [100] who reported that the systemic administration of MK886, a PPARα antagonist, did not alter anxiety-like behaviour in the OF test. The lack of significant effect of PPAR antagonism on anxiety-like behaviour is not in line with what was observed by Domi et al. (2016), who demonstrated that the administration of GW9662 had anxiogenic effects in mice in the LDB, OF, and EPM tests. However, the use of different species may explain the difference in results. To our knowledge, the present study is the first one to investigate the effects of the blockade of PPARβ/δ in anxiety responses. Both pioglitazone and rosiglitazone (PPARγ agonists) have previously been shown to elicit anxiolytic-like effects in the LDB, EPM, and OF tests [28,29,101]. Only one recent study has investigated the effect of PEA on anxiety-related behaviour and showed that chronic administration of PEA increased exploration time in the OF test, an effect blocked by the PPARα antagonist MK886 [43]. However, these authors used a higher concentration of PEA (i.e., 2.5 mg/kg) than that used in the present study and only analysed exploratory behaviour and immobility in the OF test.

Furthermore, in the present experiment, CFA-injected rats exhibited impaired recognition and spatial mnemonic performance in the NOR test. These results are in accordance with previous studies that have indicated CFA-induced cognitive deficits. Yang et al. (2014) [102] have demonstrated that CFA injection impairs the learning of tone-footshock, but not context-footshock, association in mice. Interestingly, we found that the administration of the PPARα antagonist GW6471 further impaired spatial memory in CFA-injected rats, but not in No-CFA injected controls. Importantly, the analysis of the levels of endogenous ligands at PPARs in the dorsal hippocampus conducted in the present study has shown that OEA levels were higher in CFA-injected animals compared to No-CFA counterparts. This difference in OEA levels related to inflammatory pain status may explain the differential effect of PPARα antagonism at the behavioural level. A previous study revealed that a 5-day treatment with OEA restored ethanol/THC-related decreases in both short-term spatial memory (spontaneous alternation by Y-maze) and circulating levels of BDNF in the dorsal hippocampus [84], revealing an important role for OEA in memory formation. Interpreting our results in the context of these findings, it is possible that increased OEA levels in the hippocampus may act at PPARα as a compensatory mechanism to preserve the reduced level of spatial memory function that remains following CFA injection. This may explain why pharmacological blockade of PPARα impaired spatial memory in CFA-injected rats but not in No-CFA controls. The effects of PPAR blockade on cognitive tasks is less explored. In the present study, the systemic administration of GW6471 reduced spatial mnemonic performance in the NOR test in CFA-treated rats. Additionally, the administration of PEA did not have any effect on cognitive performance. An effect of PPAR activation on learning has previously been described. Mazzola et al. (2009) [12] showed that the administration of URB597 (FAAH inhibitor) before the learning trial of a passive avoidance test enhanced the learning of the task, and this enhancement was attenuated by the administration of a PPARα antagonist MK886. These authors also demonstrated that the administration of a PPARα agonist WY14643 produced learning enhancement effects similar to those observed with URB597, which were also blocked by MK886. Also, a study from Campolongo et al. (2009) [43] indicated that the administration of OEA improved learning of passive avoidance and spatial memory tasks when given immediately post-training and that the actions of OEA were mimicked by the PPARα agonist GW7647 while being absent in PPARα null mice. Recently, Ratano et al. (2017) [44] have shown that the cognitive-enhancing effects of URB597 are dependent on PPARα, as well as on CB1 and TRPV1 receptors. Pioglitazone administration improved short-term mnemonic performance in mice, an effect most likely mediated through the PPARγ pathway [103]. However, the data herein did not observe any effect of PPARγ antagonism on object recognition memory. Furthermore, the data demonstrate a lack of effect of PEA in the NOR task, although the specific effect of PEA on cognition still needs to be explored in greater detail.

In conclusion, these results provide evidence for a modulatory effect of CFA-induced chronic inflammatory pain on cognitive processing, but not on innate anxiety-related responses. Moreover, while PPARβ/δ and PPARγ blockade or PEA did not modulate pain, cognition- or anxiety-related behaviour, blockade of PPARα exacerbated the CFA-induced impairment of spatial memory and tended to increase anxiety-related responses in the LDB test without affecting mechanical hypersensitivity. Increased OEA-PPARα signalling may act as a compensatory mechanism to preserve spatial memory function following CFA injection.

## Figures and Tables

**Table 1:** Equations for the indices used in the assessment of NOR behaviour.

Index	Day	Equation
Preference index	Familiarisation Day	[T_O3_/(T_O1_ + T_O2_/2) + T_O3_] × 100
	Test Day	[T_N_/(T_O1_ + T_O2_/2) + T_N_] × 100
Discrimination index	Test Day	[T_N_/(T_O1_ + T_O2_/2) + T_N_] × 100
Spatial Discrimination index	Familiarisation and Test Days	[(T_N_ − T_O3_)/(T_N_ + T_O3_)] × 100

T_O1_ = time exploring Object 1, T_O2_ = time exploring Object 2, T_O3_ = time exploring Object 3, and T_N_ = time exploring the new object.

## Data Availability

The data presented in this study are available on request from the corresponding author.
